# Magnetic Resonance-Guided Stereotactic Body Radiotherapy of Liver Tumors: Initial Clinical Experience and Patient-Reported Outcomes

**DOI:** 10.3389/fonc.2021.610637

**Published:** 2021-06-09

**Authors:** Fabian Weykamp, Philipp Hoegen, Sebastian Klüter, C. Katharina Spindeldreier, Laila König, Katharina Seidensaal, Sebastian Regnery, Jakob Liermann, Carolin Rippke, Stefan A. Koerber, Carolin Buchele, Jürgen Debus, Juliane Hörner-Rieber

**Affiliations:** ^1^ Department of Radiation Oncology, Heidelberg University Hospital, Heidelberg, Germany; ^2^ Heidelberg Institute of Radiation Oncology (HIRO), Heidelberg, Germany; ^3^ National Center for Tumor Diseases (NCT), Heidelberg, Germany; ^4^ Heidelberg Ion-Beam Therapy Center (HIT), Department of Radiation Oncology, Heidelberg University Hospital, Heidelberg, Germany; ^5^ Clinical Cooperation Unit Radiation Oncology, German Cancer Research Center (DKFZ), Heidelberg, Germany; ^6^ German Cancer Consortium (DKTK), Heidelberg, Germany

**Keywords:** stereotactic body radiotherapy, liver metastases, MR-guided, hepatocellular carcinoma, patient reported outcomes

## Abstract

**Purpose/Objective:**

Stereotactic body radiation therapy (SBRT) has emerged as a valid treatment alternative for non-resectable liver metastases or hepatocellular carcinomas (HCC). Magnetic resonance (MR) guided SBRT has a high potential of further improving treatment quality, allowing for higher, tumoricidal irradiation doses whilst simultaneously sparing organs at risk. However, data on treatment outcome and patient acceptance is still limited.

**Material/Methods:**

We performed a subgroup analysis of an ongoing prospective observational study comprising patients with liver metastases or HCC. Patients were treated with ablative MR-guided SBRT at the MRIdian Linac in the Department of Radiation Oncology at Heidelberg University Hospital between January 2019 and February 2020. Local control (LC) and overall survival (OS) analysis was performed using the Kaplan–Meier method. An in-house designed patient-reported outcome questionnaire was used to measure patients’ experience with the MR-Linac treatment. Toxicity was evaluated using the Common Terminology Criteria for Adverse Events (CTCAE v. 5.0).

**Results:**

Twenty patients (with n = 18 metastases; n = 2 HCC) received MR-guided SBRT for in total 26 malignant liver lesions. Median biologically effective dose (BED at α/β = 10) was 105.0 Gy (range: 67.2–112.5 Gy) and median planning target volume was 57.20 ml (range: 17.4–445.0 ml). Median treatment time was 39.0 min (range: 26.0–67.0 min). At 1-year, LC was 88.1% and OS was 84.0%. Grade I° gastrointestinal toxicity °occurred in 30.0% and grade II° in 5.0% of the patients with no grade III° or higher toxicity. Overall treatment experience was rated positively, with items scoring MR-Linac staff’s performance and items concerning the breath hold process being among the top positively rated elements. Worst scored items were treatment duration, positioning and low temperature.

**Conclusion:**

MR-guided SBRT of liver tumors is a well-tolerated and well-accepted treatment modality. Initial results are promising with excellent local control and only mildest toxicity. However, prospective studies are warranted to truly assess the potential of MR-guided liver SBRT and to identify which patients profit most from this new versatile technology.

## Background and Purpose

Surgical resection was one of the first local ablative treatment options for selected patients with hepatic oligometastases (1). In a retrospective cohort of 612 patients, resection of colorectal liver metastases led to a remarkable long-term survival of 17% after 10 years (2). However, only up to 20% of patients with hepatic oligometastases are initially amenable for surgery (3, 4). In case of reduced general condition, insufficient liver function or critical localization of the liver tumor, cryoablation, radiofrequency- and microwave ablation as well as transarterial chemoembolization are treatment alternatives for local therapy of both hepatic metastases and also primary liver tumors (5, 6). Lately, stereotactic body radiotherapy (SBRT) has been proven as a further safe and effective non-invasive treatment option (7–10).

In case of limited tumor burden, modern radiotherapy evolved from treatment of a whole organ to targeting specific lesions within the organ. In the last century, irradiation of the liver was therefore predominantly used to for palliation, due to dose limiting toxicity together with the fear of radiation-induced liver disease (RILD) (11–14). Nowadays, SBRT offers application of highly conformal tumoricidal irradiation doses whilst sparing surrounding organs at risk (OAR) due to a steep dose gradient. However, adjacent stomach, duodenum and small bowel still represent dose limiting OAR, which impede the goal of achieving ablative irradiation doses (15–18). Standard image guidance with cone beam CT scans only offers a limited soft tissue contrast impairing differentiation between tumor lesions and surrounding radiosensitive OAR (19). Additionally, respiratory motion of the liver causes anatomic changes of up to several centimeters, which can lead to inferior local control, if not adequately accounted for (20–23). Traditionally, motion management includes the usage of an internal target volume (ITV) concept resulting in larger, unnecessary target volumes which might further harm OAR (24). Advanced motion management strategies comprise gating and tracking of the target lesion: surface-guided (SG) SBRT uses the body surface as a surrogate structure for image guidance including patient positioning, intra-fraction motion monitoring and respiratory gating (25–28), while the Cyberknife system can track invasively implanted fiducials using frequent noncoplanar X-ray scans (29). MR-guided radiotherapy has recently become clinically available offering additional superior soft-tissue contrast for precise identification of liver lesions and adjacent OAR. Furthermore, some MR-Linac systems also enable gated dose delivery which offers the possibility to further reduce safety margins (30). Available literature on MR-guided SBRT for malignant liver lesions is growing, but still limited. Especially, patient acceptance needs to be evaluated, considering the long treatment time of MR-guided irradiation of the liver, which is further prolonged through online treatment adaptation (31, 32).

## Methods

The presented study is a subgroup analysis from a prospective observational trial comprising cancer patients with liver metastases or primary hepatocellular carcinoma (HCC), who were referred to our institution because they were deemed medically or functional inoperable or refused resection. Patients were treated with ablative MR-guided SBRT at the MRIdian Linac (ViewRay Inc., Mountain View, CA) in the Department of Radiation Oncology at Heidelberg University Hospital between January 2019 and February 2020. According to the guideline of the working group “Stereotactic Radiotherapy” of the German Society of Radiation Oncology (DEGRO), SBRT was defined as single fraction doses ≥ 4 Gy and number of fractions ≤12 (33).

### Treatment Characteristics

A detailed description of our treatment simulation and planning has been published previously (31). Four of our analyzed patients had already been previously included and published in this referenced study. In short, treatment simulation at the MR-Linac was performed to both acquire MR image data and to check for patients’ compliance. Three-dimensional (3D) simulation MR images, using the TrueFISP sequence (a steady-state coherent MRI sequence) with an acquisition time of 17 to 25 s were obtained in deep inspiration breath-hold, followed by planar cine-MRI in a sagittal plane to evaluate target motion characteristics (34). For the 3D simulation MRI, in-plane resolutions of either 1.5 × 1.5 mm^2^ or 1.6 × 1.6 mm^2^ and slice thicknesses of 3 mm with varying fields-of-view were used. No MR contrast fluid was administered. The acquired MR image data was used as the primary image set for treatment planning. All patients received additional diagnostic, contrast-enhanced MRIs for treatment planning. Furthermore, a planning CT scan with and without contrast enhancement was performed to also obtain data on electron density information for dose calculation. The gross tumor volume (GTV) was delineated as the macroscopic tumor volume on all available co-registered planning imaging modalities, with a clinical target volume (CTV) expansion of 5 mm and additional 3 mm for creating the planning target volume (PTV) due to technical uncertainties.

Daily image guidance was performed for each fraction by onboard 3D MRI using identical settings (field of view, duration, pulse sequence, breathing instructions) as during MR simulation. Soft-tissue based registration with the reference MR scan was applied, always registered directly on the GTV.

Gated dose delivery in breath hold was performed. The TrueFISP sequence was applied for real time MR-gating (cine-MRI scan) within one sagittal slice and four frames per second. If the liver lesion was visible on the TrueFISP sequence, the lesion was used as the gating structure (region of interest; ROI). This was the case in 14 of the 20 analyzed patients. Otherwise, an anatomical surrogate structure in proximity of the target lesion was defined as the gating target. In five patients, the nearest surface of the respective liver segment was used for this purpose. In one patient, a prominent adjacent liver vessel was defined as the surrogate structure. The predefined ROI (either the GTV or the surrogate structure) was expanded by 3 mm in every direction, which formed the gating boundary. The irradiation beam was automatically shut off, if the target structure (usually the GTV) left the gating boundary, including a tolerance threshold of mostly 3%, with a maximum of 7% in very rare cases. During gated dose delivery, patients were offered visual guidance *via* an in-room monitor displaying the live sagittal cine-MR image with an overlay of the gating target and the boundary. A video of this process can be found in the supplementary material section. If an intrafractional GTV deviation occurred and the patient could therefore no longer keep the ROI within the boundary, a table correction including a subsequent new MRI scan had to be performed. This procedure was mandatory to allow for a 3D table correction, since the cine-MRI only provides a 2D image control (in the sagittal plane). No online treatment adaptation was performed, as this technique had not yet been implemented, when the patients were treated.

Doses and fractionation schemes depended on the size and localization of the hepatic lesion as well as patients’ breath holding capability. In general, small and centrally located lesions were treated with three fractions of 15 Gy, prescribed to the conformally enclosing 65% isodose, while larger lesions (>5 cm) were irradiated with eight fractions of 7.5 Gy or five fractions of 10Gy prescribed to the conformally enclosing 80% isodose. Hepatic lesions in close proximity to radiosensitive OAR were usually treated with ten fractions of 5 Gy prescribed to the conformally enclosing 80%-isodose. One hepatic metastasis was treated with twelve fractions of 4 Gy prescribed to the conformally enclosing 95% isodose as the patient had been treated with prior hemihepatectomy and the lesion was diagnosed at the liver margin which had been sutured to the small bowel.

Target coverage was comprised if required OAR dose constraints could not be met. Applied dose constraints were the following (for five fractions):

−esophagus: 0.5 cc <34 Gy
−stomach/intestine: 0.5 cc <35 Gy
−liver minus CTV: ≥700 cc <24 Gy
−kidney: mean dose <10 Gy
−spinal cord 0.1 cc <27 Gy
−heart: 0.5 cc <29 Gy.

An in-house designed patient-reported outcome questionnaire (PRO-Q) was used to measure patients’ experience with the MR-Linac treatment (grades from 1–5, where 1 represents a completely positive and 5 a completely negative experience) (31). Patients were additionally asked, how many minutes it took to fully mentally and physically recover after their effort during the respective treatment session. Furthermore, our staff was surveyed about their opinion on each patient’s treatment performance (grades from 1–10, where 1 represents a completely easy and 10 an almost inacceptable expenditure).

### Endpoints and Statistical Methods

LC and OS were estimated starting from the first day of the SBRT. LC was calculated based on each lesion, whereas OS was calculated per patient. The Response Evaluation Criteria in Solid Tumors (RECIST 1.1) was used to asses tumor response. Toxicity was described using the Common Terminology Criteria for Adverse Events (CTCAE v. 5.0).

In accordance with the study protocol, each patient was specifically assessed for presence of fatigue, nausea, vomiting, diarrhea, constipation, dyspnea, cough, skin disorder and pain. This evaluation was performed before irradiation, at the last treatment day and at first follow-up. Follow-up consisted of a contrast fluid enhanced MRI or CT scan of the liver, performed six to eight weeks after completion of the SBRT together with a clinical examination. Further imaging follow-up was performed every three months afterwards and consisted of a contrast fluid enhanced CT of the thorax and the abdomen or a contrast-enhanced MRI, but was not part of the prospective study. The Child–Pugh score was assessed within four weeks prior to the SBRT and at the first follow-up examination.

LC and OS were estimated using the Kaplan–Meier method. The biologically effective dose (BED) was calculated applying the linear-quadratic model (35). An α/β ratio of 10 was assumed for liver metastases and HCC.

BED(Gy)=single dose×number of fractions(1+single doseα/β)

All statistical analyses were performed with SPSS software (IBM SPSS Version 24.0). A p-value of <0.05 was defined significant. The MR-Linac observational study was approved by the Ethics committee of the University Hospital Heidelberg (S-543/2018). Written informed consent was obtained from all patients included into the study.

### Two Selected Cases From Daily Routine

For providing detailed clinical inside into treatment reality at the MR-Linac, two characteristic patients were selected for in-detail description. Since gated dose delivery in breath hold is challenging, as it demands a certain amount of treatment compliance and the bore of the MR-Linac is relatively narrow (70 cm), the oldest patient and the patient with the highest body mass index were selected for further description.

## Results

Patient characteristics are described in **Table 1**. Median age of the 20 patients was 61 years. Most patients had a very good performance status and a non-obese body mass index. Most irradiated liver lesions were metastases from colorectal carcinoma. Two patients suffered from HCC. Systemic therapy was administered in most patients before (75%) and after (55%) radiotherapy. One patient underwent hemihepatectomy prior to radiotherapy. Twelve patients had already complained of grades I–II° adverse events before starting hepatic SBRT, mostly grade I° fatigue.

**Table 1:** Patient characteristics (n = 20).

**median age**	61 years	range 37–78 years
**median Karnofsky Score**	90%	range 70–100%
**median Body Mass Index**	23.8 kg/m²	range 18.0–42.3 kg/m²
**female/male**	10/10	50.0%/50.0%
**Hepatocellular Carcinoma**	2	10.0%
**Metastases**	18	90.0%
n = 5 colorectal carcinoma; n = 4 breast cancer; n = 3 malignant melanoma; n = 1 adenoid cystic carcinoma; n = 1 cholangiocellular carcinoma; n = 1 urinary bladder carcinoma; n = 1 papillary carcinoma; n = 1 pancreatic cancer; n = 1 prostate cancer.		
**distant metastases present (apart from the irradiated liver metastases)**		
**n = 0**	9	45.0%
**n = 1–5**	5	25.0%
**n > 5**	6	30.0%
prior hemihepatectomy	1	4.7%
median Child–Pugh-Score^1^	5	range 5–7
extrahepatic disease progression within four weeks before irradiation	2	10.0%
**systemic therapy within 4 weeks before irradiation**	15	75.0%
n = 10 chemotherapy; n = 3 checkpoint inhibition; n = 2 hormonal therapy		
systemic therapy within 4 weeks after irradiation	11	55.0%
n = 5 chemotherapy; n = 3 checkpoint inhibition; n = 2 hormonal therapy; n = 1 targeted therapy		
**Adverse events before radiotherapy**		
I°	10	50.0%
n = 6 fatigue; n = 2 fatigue + diarrhea; n = 2 pain + cough		
II°	2	10.0%
n = 1 nausea; n = 1 fatigue		
≥III°	0	0
**Adverse events at last treatment day**		
I°	12	60.0%
n = 5 fatigue; n = 1 nausea; n = 1 diarrhea + nausea + fatigue; n = 1 fatigue + dysphagia +erythema; n = 1 flatulence + fatigue; n = 1 fatigue + dyspepsia; n = 1 fatigue + nausea; n = 1 fatigue + dizziness		
II°	1	5.0%
n = 1 fatigue + diarrhea		
≥III°	0	0
**Adverse events at first follow-up**		
I°	8	40.0%
n = 3 fatigue; n = 1 pain; n = 1 fatigue + nausea; n = 1 nausea + diarrhea; n = 1 nausea + dyspepsia; n = 1 fatigue + pain		
II°	0	0
≥III°	0	0

^1^available data for n = 15 patients.

Most patients were treated with hepatic SBRT for one single liver lesion (n = 18), while two patients had four lesions irradiated. Median PTV size was 57.2 ml (17.4–445.0 ml). Median dose was 50 Gy (45–60 Gy) with a calculated BED of 105.0 Gy (67.2–112.5 Gy).

**Figure 1:**
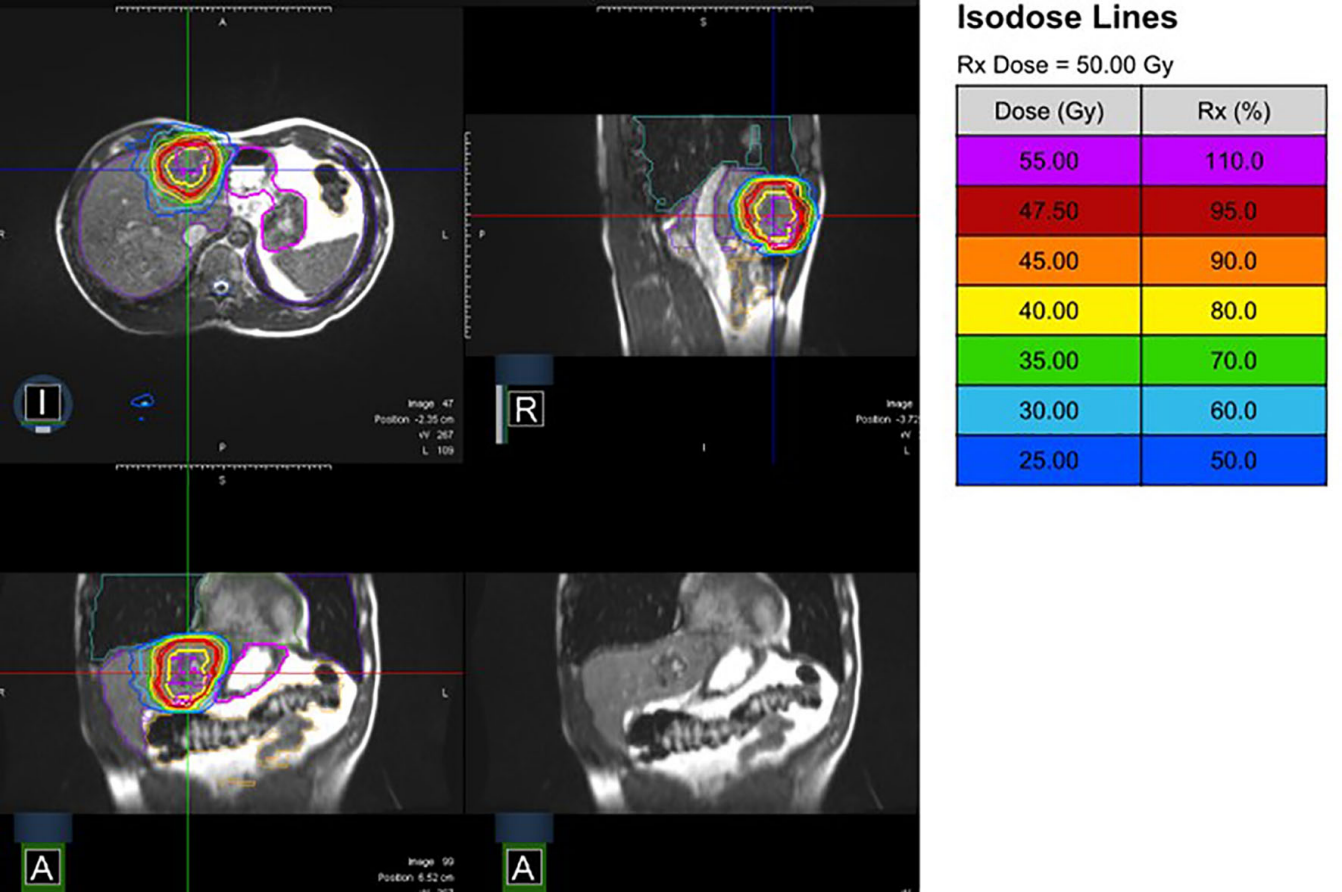
MR-Linac treatment plan (10 fractions of 5 Gy prescribed to the conformally enclosing 80%-isodose) from different perspectives (I, inferior; A, anterior; R, right) with and without isodose lines.

Further treatment characteristics are listed in **Table 2**. **Figure 1** shows a characteristic treatment plan, where maximum sparing of the neighboring small bowl and stomach could be achieved.

**Table 2:** Irradiation treatment characteristics.

total number of irradiated liver targets per patient		
**n = 1**	18	90.0%
**n = 4**	2	10.0%
**localization of liver targets**	Segment I (7.7%) II (23.1%) III (0%) IV (15.3%) V (7.7%) VI (7.7%) VII (23.1%) VIII (15.4%)	
**response to irradiation in first follow-up examination**		
**partial remission**	15	57.6%
**stable disease**	11	42.4%
	median	range
**largest axial diameter**	21 mm	8–77 mm
**GTV**	15.5 mL	1.4–255.0 mL
**CTV**	36.4 mL	6.4–349.3 mL
**PTV**	57.2 mL	17.4–445.0 mL
**prescribed total dose**	50 Gy	45–60 Gy
**fractions**	8	3–12
**dose inhomogeneity**	80%	65–80%
**EQD2(α/β = 10)**	87.5 Gy	56.0–93.8 Gy
**BED (α/β = 10)**	105.0 Gy	****67.2–112.5 Gy
**monitor units per fraction**	2,403.9	1,155.4–6,309.7
**number of beams that are on**	11	7–15
**duration of the session (“on table”)**	39.0 min	26.0–67.0 min
**-radiation time**	15.8 min	10.3–38.2 min
**-pure beam on time**	3.8 min	1.83–10.0 min

BED, biologically effective dose; CTV, clinical target volume; EQD2, equivalent dose at 2 Gy; GTV, gross tumor volume; PTV, planning target volume.

### Outcome

**Figure 2:**
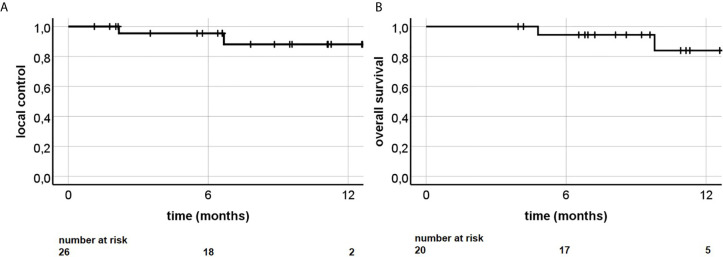
**(A)** Local control and **(B)** overall survival following MR-guided liver SBRT.

**Figure 3:**
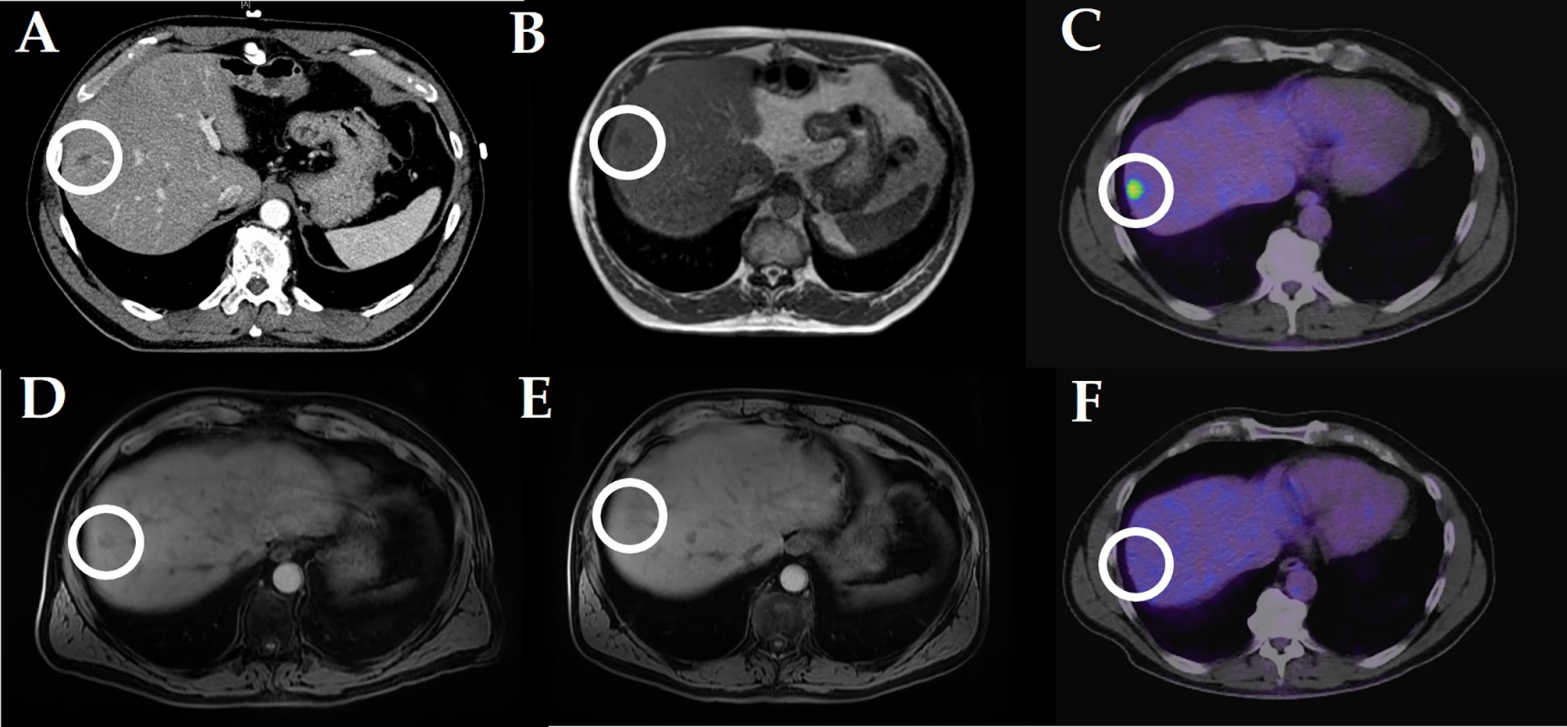
Stereotactic MR guided radiotherapy of a hepatic metastasis in a patient with pancreatic cancer (10 × 5 Gy): **(A)** planning CT scan (portal venous phase); **(B)** online liver simulation at the MR-Linac; **(C)** pre-radiotherapy FDG-PET CT scan; **(D)** first (3 months after radiotherapy) post-radiotherapy MRI scan (liver imaging with volume acceleration-flexible MRI); **(E)** second (4 months) post-radiotherapy MRI (liver imaging with volume acceleration-flexible MRI); **(F)** post-radiotherapy (4 months) FDG-PET CT scan. Comment: later hemihepatectomy revealed complete remission of the radiated liver metastasis.

Median follow-up was 9.4 months. Estimated local control was 88.1% at 12 months (**Figure 2A**). All irradiated liver lesions were stable or had a decrease in size at first follow-up (**Table 2**). Two patients (10%) died during follow-up time. Estimated OS at 12 months was 84.0% (**Figure 2B**). Child–Pugh score (available for n = 15 patients) did not decrease after irradiation. **Figure 3** illustrates a representative patient case, where additional pre- and post-radiotherapy FDG PET-CT scans were performed, which revealed only residual activity of the liver metastasis after MR-guided hepatic SBRT. Later hemihepatectomy due to disease progression in the right liver lobe outside the irradiated area revealed complete pathological remission of the irradiated lesion.

### Toxicity

Acute toxicity was mild with thirteen patients describing grades I–II° adverse events on the last day of radiotherapy, including mostly grade I° fatigue. Six patients suffered from grade I° gastrointestinal side effects and one patient was diagnosed with grade II° gastrointestinal side effects (diarrhea). Eight patients complained of grade I° adverse events at first follow-up examination, mainly fatigue. No grade III° adverse event ore higher was reported at any time.

### Patient and Staff Reported Outcome

**Table 3** displays the personal subjective experience of the treated patients. Overall treatment experience was rated positively, with items scoring MR-Linac staff’s performance and items concerning the breath hold process being among the top positively rated items (each median 1 point). Worst scored elements were treatment duration, positioning and temperature of body parts (each median 3 points). The whole treatment processes, including breathing instructions, were challenging for some patients, both mentally and physically. Median time to full mental and physical recovery after the first treatment session was 20 min (range 0–360 min). Median complexity of radiotherapy at the MR-Linac was rated as average by the staff (**Table 3**).

**Table 3:** Patient (positions 1–18) and staff (position 19) reported outcome (available for n = 18 patients).

	categorical point scale form 1–5, where 1 equals very positive and 5 equals very negative	
	median	range
**1. Overall treatment experience**	2	1–5
**2. Information provided by the staff**	1	1–5
**3. Friendliness of the staff**	1	1–5
**4. Duration of the treatment**	3	1–5
**5. Size of the MRI bore**	2	1–5
**6. Positioning during radiotherapy**	3	1–5
**7. Having to lie still**	3	1–5
**8. Noise in the MR-Linac**	2	1–4
**9. Temperature in the MR-Linac**	3	1–5
**10. Local temperature of body parts**	3	1–5
**11. Tingling sensations in fingers and toes**	2	1–5
**12. Breathing instructions**	1	1–5
**13. Breath holding**	2	1–5
**14. Anxiousness during treatment session**	1	1–5
**15. Reported time until full mental and physical recovery after the radiotherapy session**	20 min	0–360 min
**16. Difficulty to hold the target with one´s own breath**	1	1–4
**17. Ability to watch one´s own treatment *via* monitor**	1	1–2
**18. Feeling of having active control over the treatment duration**	1	1–3
**19. Treatment complexity from the perspective of the staff**	categorical point scale form 1–10, where 1 equals very positive and 10 equals very negative	
****	5	2–10

### Two Selected Cases From Daily Routine

The most obese patient (187 cm, 148 kg, body mass index = 42 kg/m²; ventrodorsal abdominal diameter = 35 cm; 52 years, Karnofsky Performance Score 70%) was treated for a single liver metastasis of a rectum carcinoma (three fractions of 15 Gy). The duration of the treatment session (40 min; “on table”, including patient positioning), was comparable to the study median (39 min). Mere radiation time (16 min) was below the median of the study cohort (22 min). Recovery time after radiotherapy (10 min) was below the study median of 20 min. No patient reported outcome item was rated worse than average. MR-Linac staff documented average complexity of the irradiation process.

The oldest patient (78 years; Karnofsky Performance Score 80%; body mass index = 30 kg/m², no reported lung disease) was treated for a single liver metastasis of a cholangiocellular carcinoma (three fractions of 15 Gy). Duration of the treatment session (38 min) and pure radiation time (21 min) were comparable to the median of the study cohort (39 and 22 min). Recovery time after radiotherapy (90 min) was more than four times the study median of 20 min. Patient reported outcome items were among the worst of the study population (treatment duration rated with 5; breath holding rated with 4). MR-Linac staff documented maximum complexity of the irradiation process.

## Discussion

In this subgroup analysis of a prospective observational study, 20 patients received MR-guided SBRT for in total 26 malignant liver lesions at Heidelberg University Hospital from January 2019 to February 2020. MR-guided SBRT for tumors in the abdomen was described to be safe in a phase-I study as well as in a study by Hal et al. with no higher-grade toxicities (36, 37). However, these studies included patients with different abdominal malignancies. Experience with MR-guided radiotherapy of malignant lesions of the liver is growing, yet still scarce (**Table 4**). Gani et al. published one of the first prospective studies investigating the MR-guided liver SBRT using a high-field MR-Linac (32). Patient acceptance was high with very low toxicity burden. As far as described, treatment toxicity was rather low in all larger studies in the field. Only two grade III° toxicities were described by Rosenberg and colleagues (40). No grade III° toxicity was reported in our study cohort, even though patients were prospectively evaluated for side effects.

**Table 4:** Studies on radiotherapy of liver lesions with a magnetic resonance imaging linear accelerator.

	patients, characteristics, design	radiation technique	Toxicity	LC	OS
Feldman et al. (38)	patients: n = 29 (n = 26 HCC, n = 2 cholangiocarcinoma, n = 1 metastatic colon cancer) irradiated lesions n = 34 median age: NA gender: NA Child–Pugh-Class: NA retrospective design	MRIdian Linac (ViewRay, Oakwood Village, OH) 0.35 T step-and-shoot IMRT; utilization of gating 27–50 Gy prescribed to at least 95% of the PTV in three or five fractions PTV margin: 5 mm mean number of beams: 10.8 (range 6–16) adaptive technique: n = 1 (3.4%) average treatment time: 34 min beam-on time: NA mean monitor units per fraction: 2,538.9 (range 1,549.1–5,737.4) median PTV volume: NA	constraints: American Association of Physicists in Medicine Task Group 101 (39) general toxicity: n = 1 nausea and vomiting n = 1 abdominal pain with bloody diarrhea (n = 4 deaths due to liver cirrhosis, unrelated to radiation effect)	–	–
Rosenberg et al. (40)	patients: n = 26 (n = 8 colorectal adenocarcinoma, n = 6 HCC, n = 3 lung, n = 2 cholangiocellular, n = 1 pancreas, n = 1 sarcoma, n = 1 head and neck, n = 4 others) liver lesions present: 1–3 per patient median age: 70 y (30–90 y) female: n = 9 (35%) Child–Pugh-Class: A (76,9%); NA (23,1%) retrospective design	MRIdian System (ViewRay Inc., Mountain View, CA) 0.35 T MRI scanner combined with 3 co-planar cobalt sources; utilization of gating median dose 50 Gy (range 30–60 Gy) in five fractions (6–12 Gy/fraction) PTV margin: 2–5 mm number of beams: 12–15 adaptive technique: no range of treatment time: 40–60 min range of beam-on time: 20–30 min occasional use of gadoxetic acid 20 min before treatment as contrast fluid median PTV volume: 98.2 cm³ (13–2,034 cm³)	constraints: –mean liver dose: <13 to 15 Gy, >700 cm³ of liver receiving less than 15 Gy (liver-GTV), –stomach and bowel: V32–33 <0.5 cm³ gastrointestinal toxicity: I–II°: NA III°: 7.7% ≥IV°: 0% n = 2 decrease in Child–Pugh-Class n = 1 significant hilar stricture n = 1 portal hypertension	80,4%@21m (100% in case of HCC)	69.0% @ 1 y
Gani et al. (32)	Patients: n = 10 (metastases of n = 5 colorectal adenocarcinoma, n = 1 esophageal, n = 1 melanoma, n = 1 cystic duct, n = 1 GIST, n = 1 ACC) no patients with Child B or Child C cirrhotic liver disease median age: 68 y (48–86 y) female: n = 5 (50%) sub-study of a basket phase 2 feasibility trial (NCT04172753)	1.5 T MR-Linac (Unity, Elekta, Crawley, UK) median dose 38.5 Gy to 98% of the GTV internal target volume concept PTV margin: 3–6 mm adaptive technique: yes range of treatment time: 26 -36min median beam-on time: 9.6min median PTV volume: 96.2 cm³ (11.3-399.5cm³)	constraints: based on the UKSABR guidelines (Version 6.1) no increase in transaminases >I° or gastrointestinal toxicity with the necessity of medical intervention	–	–
Rogowski et al. (41)	Patients: n = 11 Lesions: n = 15 (n = 2 Cholangiocarcinoma; metastases of n = 6 neuroendocrine tumor, n = 4 colorectal adenocarcinoma, n = 2 sarcoma, n = 1 GIST) median age 66 y (47–86 y) female: n = 5 (46%) prospective observational clinical trial	0.35T hybrid MR-Linac (Viewray Inc., Mountain View, CA, USA) mainly 12.5 Gy in three fractions PTV margin: 3–5 mm adaptive technique: yes median treatment time: 53 min median beam-on time: 10 min median PTV volume: 39.1 cm³ (8.3–411.3 cm³)	toxicity: I°: 55% ≥II°: 0%	100% @me-dian follow-up of 5 m	–
Weykamp et al. (present study)	Patients: n = 20 (n = 2 HCC; metastases of: n = 5 colorectal, n = 4 breast cancer, n = 3 melanoma, n = 1 adenoid cystic carcinoma, n = 1 cholangiocellular carcinoma, n = 1 urinary bladder, n = 1 papillary carcinoma, n = 1 pancreatic cancer, n = 1 prostate cancer) median age: 61 y (37–78 y) female: n = 10 (50%) Child–Pugh-Class: A (70,0%); B (5%); NA (25,0%) subgroup analysis of a prospective observational study	MRIdian Linac (ViewRay, Oakwood Village, OH) 0.35 T step-and-shoot IMRT; utilization of gating median dose 50 Gy (range 45–60Gy) in eight fractions (3–12 Gy/fraction) CTV margin 5 mm PTV margin: 3 mm number of beams: 7–15 adaptive technique: no median duration of the session (“on table”): 39.0 min (26.0–67.0 min) median radiation time: 15.8 min (10.3–38.2 min) median monitor units per fraction: 2,403.9 (1,155.4–6,309.7) no MRI contrast fluid median PTV volume: 57.2 cm³ (17.4–445.0 cm³) median liver dose: 12.7 Gy (3.2–21.9 Gy)	constraints (for five fractions): –esophagus: 0.5 cc <34 Gy –stomach/intestine: 0.5 cc <35 Gy –liver minus CTV: ≥700 cc <24 Gy –kidney: mean dose <10 Gy –spinal cord 0.1 cc <27 Gy –heart: 0.5 cc <29 Gy gastrointestinal toxicity: I°: 30.0% II°: 5.0% ≥III°: 0% n = 0 decrease in Child–Pugh-Class	88.1% @ 1y	84.0% @ 1y

ACC, adenoid cystic carcinoma; GIST, gastrointestinal stroma tumor; HCC, hepatocellular carcinoma; m, months; min, minute; mm, millimeter; MRI, magnetic resonance imaging; NA, not available; y, years.

The higher proportion of patients with HCC in the two US-American studies can be explained by epidemiology as well as the higher prevalence of viral hepatitis and obesity compared to Germany (38, 40, 42). Furthermore, in our study, estimated LC was excellent, with 88% at 1 year. However, with a median of 9.4 months, follow-up of our cohort is still rather short. One of the previously mentioned US-American studies provided data on treatment outcome: Rosenberg et al. reported a LC of 80% at the median follow-up of 21.2 months (40). Furthermore, Rogowski et al. described a local control rate of 100%, however with a median follow-up of 5 months (41). Preliminary LC results are therefore so far comparable to non-MRI-guided liver SBRT, as recently described in a systematic review by Ohri et al. with a LC after 1- and 2-years of 90 and 79% (43). Our estimated 1-year OS was higher than described by Rosenberg et al. (84% *vs*. 69%) and might be explained by the younger median age in our cohort (61 years *vs*. 70 years). The proportion of different primary tumors (mainly colorectal) was comparable as well as the median prescribed irradiation dose (**Table 4**). Nonetheless, Rosenberg et al. used cobalt sources instead of a linear accelerator. Furthermore, median PTV was nearly half the size as in our study cohort (38). Future follow-up will show, whether these circumstances will lead to a differing LC or OS.

An essential part of improving treatment quality at the MR-Linac is to assess patients’ perspectives. Wearing headphones in an MRI scanner is a common and easy procedure to cope for the operating noise. This procedure seems to be sufficient in our study cohort as reflected by the positive patient reported outcome, in contrast to the room temperature. For optimal functionality of both MRI scanner and linear accelerator, the room temperature is leveled down. Moreover, to reduce the risk of metal items being accidentally taken into the vicinity of the magnetic field, patients wear hospital provided medical scrubs during the irradiation sessions, which are rather thin. Both circumstances explain the negative patient reported results concerning the temperature. As a reaction to our study results, we began to ask patients immediately before the irradiation session, whether they tend to feel cold easily. If so, patients are provided with additional blankets.

Surprisingly, patients were not disturbed by their own tumor being displayed on a monitor. Correspondingly, our data reveal that the breath hold procedure as a whole is perceived very positively by the patients. A more difficult terrain for improvement is patient positioning, treatment duration and the fact that patients have to lie still on the treatment couch for a relatively long time. Devices for patient immobilization are more challenging to be developed for the MR-Linac because they have to be both non-magnetic and adequate for the rather small bore (44). Treatment duration and not being allowed to move were perceived rather negative. Our practice to play radio music to the patients *via* headphones seems not to be sufficient to guarantee full patient comfort. One must keep in mind, that our presented patient cohort had been treated before daily online plan adaption was implemented at our MR-Linac, which surely further prolongs treatment duration. Patient positioning and MR-imaging procedure consume a large amount of time. Less than half of the treatment session is used for the irradiation process itself (including the gating procedure). Beam-on time even accounts for less than a tenth of the treatment duration (**Table 2**).

Based on the benefits mentioned above, The Lancet Oncology recently dedicated a whole review to the high capability of MR-guided liver SBRT. Witt and colleagues emphasized the potential of MR-guided adaptive SBRT to become a practice changing technology for irradiation of the liver (44). However, radiotherapy with the MR-Linac is resource intensive in terms of personnel, time, money and required patient compliance. Hence, it is of the utmost importance to identify the ideal patients for receiving MR-guided SBRT. To date, three prospective trials are going to investigate the potential of online adaptation in SBRT for liver malignancies. An US-American phase-I study aims to reveal the safe maximum tolerated dose for MR-guided SBRT treatment liver metastases through real time adaptation (45). The French phase-II RASTAF study will investigate Adaptative MR-Guided Stereotactic Body Radiotherapy of Liver Tumors (46). Our planned phase-II MAESTRO trial (magnetic resonance-guided stereotactic radiotherapy for hepatic metastases) is going to evaluate, if a higher proportion of liver lesions can be treated with locally ablative doses of a biologic effective dose ≥100 Gy when applying MR-guided adaptive compared to standard ITV-based-SBRT.

The main limitation of the presented study is its small sample size. It was statistically not possible to detect factors which estimate patient acceptance. Instead, we provided a detailed description of the oldest patient and the patient with the highest body mass index. Obesity led to average treatment expenditure as rated by patient and staff. On the contrary, the oldest patient reported the worst negative scores, which was in accordance with the judgement by the staff. This might be explained by the exhausting breathing commands while observing the monitor carefully, leading to a demanding multitasking treatment environment. As a rule of thumb, patients at our clinic are asked if they can hold their breath for at least 25 s and whether they can picture themselves repeating this breath-holding several times whilst lying on a non-padded surface for about an hour. The evaluation of both the friendliness of our staff and the treatment expenditure appear to be highly subjective question items. However, since treatment at the MR-Linac is complex and demanding for the patient, guaranteeing an environment of thrust is highly important to ensure compliance, especially when breathing instructions are involved. Furthermore, the subjective rating of the treatment expenditure will help to identify patient characteristics, which may disqualify patients for treatment at the MR-Linac in the first place. Follow-up was rather short. Since the majority of radiation-induced liver diseases occur within the first three to four months after treatment, long-term toxicity might be underestimated to a certain degree (12).

We demonstrated that MR-guided SBRT of liver malignancies is a resource intensive treatment method both for the patient and the radio-oncology department. Further follow-up will reveal whether MR-guided SBRT will significantly improve clinical results compared to conventional techniques. Using the body surface as a surrogate parameter for image guidance, SG-SBRT might be even faster and more convenient than MR-guided SBRT. Albeit, the movement of the liver is not directly monitored (25–27) and the correlation of skin to tumor is not always constant especially for liver and pancreatic tumors (47, 48). Furthermore, Stick et al. investigated intrafractional fiducial marker position variations during visually guided, deep-inspiration breathhold (DIBH) SBRT of liver metastases and reported deviations of up to 10 mm. Based on those findings, the colleagues concluded that for ensuring accurate dose delivery real-time monitoring during treatment, e.g. MR-guided SBRT, is necessary and now apply MR-guided radiotherapy for liver metastases (49). Another option might be the additional application of ultrasound monitoring applied during SBRT with active breathhold control, which has been reported to reduce residual motion to <5 mm in most cases (50). Fiducials enable the Cyberknife system to directly track the immediate treatment area, however fiducial placement is an invasive procedure, which can cause liver trauma, bleedings or a pneumothorax (51, 52). Furthermore, treatment duration of hepatic SBRT applying tumor tracking at the Cyberknife might also easily exceed 30 min. MR-guided hepatic SBRT offers a non-invasive treatment alternative with direct intrafractional visualization of the tumor hereby ensuring optimal target coverage.

Since late February 2020, our clinic has been using online adaptation. Daily SBRT treatment can now be prescribed to the anatomy of the day, taking into account interfractional and even intrafractional changes, due to organ motion (53–56). Therefore, OAR can be superiorly spared and higher irradiation doses can be achieved (36, 40, 54). However, online adaptation further prolongs the duration of the treatment session and has already needed to be omitted in a few cases during our first clinical experience to secure compliance.

We showed that MR-guided SBRT is safe and effective, even without online adaptation. It might be especially adequate for selected patients with liver malignancies very close to OAR who refuse the invasive placement of fiducials.

## Conclusion

We demonstrated that MR-guided SBRT of malignant liver lesions is a well-tolerated and well-accepted non-invasive treatment modality with only mild toxicity. Moreover, we provided insights into patient reported outcomes, which might support patient selection for this highly promising but nonetheless resource intensive treatment modality.

## Data Availability Statement

The datasets generated for this study are available on request to the corresponding author.

## Ethics Statement

The studies involving human participants were reviewed and approved by the Ethics Committee of the University Hospital Heidelberg (S-543/2018). The patients/participants provided their written informed consent to participate in this study.

## Author Contributions

FW performed the data collection and the statistical analysis and drafted the manuscript. SAK, PH, LK, KS, SR, JL, and SK helped with data collection as well as figure and table preparation. SK, CS, CR, and CB performed treatment planning and contributed the medical physicist expertise. JH-R and JD participated in the study design and helped to draft the manuscript. All authors contributed to the article and approved the submitted version.

## Funding

SR and JL are funded by the Physician-Scientist Program of Heidelberg University, Faculty of Medicine.

## Conflict of Interest

JH-R received speaker fees and travel reimbursement from ViewRay Inc., as well as travel reimbursement form IntraOP Medical and Elekta Instrument AB outside the submitted work. JD received grants from CRI—The Clinical Research Institute GmbH, View Ray Inc., Accuray International, Accuray Incorporated, RaySearch Laboratories AB, Vision RT limited, Astellas Pharma GmbH, Merck Serono GmbH, Astra Zeneca GmbH, Solution Akademie GmbH, Ergomed PLC Surrey Research Park, Siemens Healthcare GmbH, Quintiles GmbH, Pharmaceutical Research Associates GmbH, Boehringer Ingelheim Pharma GmbH Co, PTW-Freiburg Dr. Pychlau GmbH, Nanobiotix A.A. as well as IntraOP Medical outside the submitted work. SK has received personal fees and travel reimbursement from Viewray.

The remaining authors declare that the research was conducted in the absence of any commercial or financial relationships that could be construed as a potential conflict of interest.
